# Capgras and Fregoli delusions - a case report

**DOI:** 10.1192/j.eurpsy.2022.1813

**Published:** 2022-09-01

**Authors:** M. Viseu, F. Tavares, M. Barbosa Pinto

**Affiliations:** University Hospital Center of Algarve, Portugal, Department Of Psychiatry And Mental Health – Faro, Faro, Portugal

**Keywords:** Delusional Misidentification Syndromes, Capgras delusion, Fregoli delusion

## Abstract

**Introduction:**

Capgras and Fregoli delusions are psychotic complex conditions that integrate a larger group of Delusional Misidentification Syndromes (DMS), where the patient misidentifies people, places or objects.

**Objectives:**

Review of the literature and exposure of a case report of a first psychotic episode with DMS.

**Methods:**

Case report and a nonsystematic review through databases. With the keywords: Capgras; Fregoli; DMS.

**Results:**

This case presents a caucasian male, 34-year-old, with no personal or family history of psychiatric pathology. He was taken to the emergency service, due to behavior disorder with verbal aggressiveness to his mother. During the psychiatric emergency he was restless, with accelerated speech and presented Capgras and Fregoli delusion. He believed that his mother had been replaced by an imposter and that the doctor was actually his childhood friend disguised, all with the intention of harming him. The patient was hospitalized, analytical and brain scan show no abnormally. Improvement in symptoms was been seen when a long-term injectable antipsychotic was started. The diagnosis was Bipolar type I disorder.

**Conclusions:**

DSM are more frequent than previously considered, they often occur in association with psychiatric or neurological disorders. Case reports like this one helps to clarify the association between DMS and psychiatric disorders. Given the high incidence of DMS, it is essential to recognize them, carry out an early treatment and be alert to other psychopathological or neurological symptoms that may coexist.

**Disclosure:**

No significant relationships.

